# Optimizing ventricular tachycardia ablation through imaging-based assessment of arrhythmic substrate: A comprehensive review and roadmap for the future

**DOI:** 10.1016/j.hroo.2024.07.001

**Published:** 2024-07-05

**Authors:** Janneke C. Burger, Luuk H.G.A. Hopman, Michiel J.B. Kemme, Wiert Hoeksema, Richard A.P. Takx, Rosa M. Figueras I Ventura, Fernando O. Campos, Gernot Plank, R. Nils Planken, Cornelis P. Allaart, Vokko P. van Halm, Pieter G. Postema, Marco J.W. Götte, Martin J. Bishop, Pranav Bhagirath

**Affiliations:** ∗ Department of Cardiology, Amsterdam University Medical Center, Amsterdam, The Netherlands; † Department of Radiology and Nuclear Medicine, Amsterdam University Medical Center, Amsterdam, The Netherlands; ‡ Adas3D Medical S.L., Barcelona, Spain; § School of Biomedical Engineering and Imaging Sciences, King’s College London, London, United Kingdom; ¶ Gottfried Schatz Research Center, Division of Biophysics, Medical University of Graz, Graz, Austria

**Keywords:** Ventricular tachycardia, Image integration, Arrhythmogenic substrate, Cardiac magnetic resonance imaging, Cardiac computed tomography

## Abstract

Ventricular tachycardia (VT) is a life-threatening heart rhythm and has long posed a complex challenge in the field of cardiology. Recent developments in advanced imaging modalities have aimed to improve comprehension of underlying arrhythmic substrate for VT. To this extent, high-resolution cardiac magnetic resonance (CMR) and cardiac computed tomography (CCT) have emerged as tools for accurately visualizing and characterizing scar tissue, fibrosis, and other critical structural abnormalities within the heart, providing novel insights into VT triggers and substrate. However, clinical implementation of knowledge derived from these advanced imaging techniques in improving VT treatment and guiding invasive therapeutic strategies continues to pose significant challenges. A pivotal concern lies in the absence of standardized imaging protocols and analysis methodologies, resulting in a large variance in data quality and consistency. Furthermore, the clinical significance and outcomes associated with VT substrate characterization through CMR and CCT remain dynamic and subject to ongoing evolution. This highlights the need for refinement of these techniques before their reliable integration into routine patient care can be realized. The primary objectives of this study are twofold: firstly, to provide a comprehensive overview of the studies conducted over the last 15 years, summarizing the current available literature on imaging-based assessment of VT substrate. Secondly, to critically analyze and evaluate the selected studies, with the aim of providing valuable insights that can inform current clinical practice and future research.

## Graphical abstract

**Figure:**
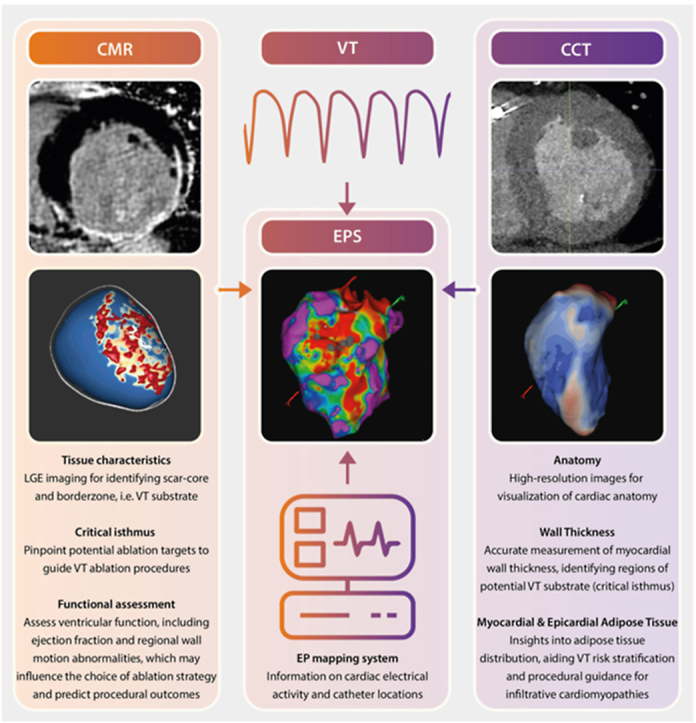

**Key Findings**

▪ Both cardiac magnetic resonance (CMR) imaging and cardiac computed tomography (CCT) are valuable for identifying ventricular tachycardia (VT) substrate, and their integration with electroanatomic mapping (EAM) systems significantly improves the precision and efficacy of VT ablation procedures. This combination allows for comprehensive assessment and targeted ablation of scar-related VT.
▪ High-resolution 3-dimensional CMR imaging, particularly with gadolinium enhancement, enhances the detailed visualization of scar tissue, border zones, and conduction channels. This advanced imaging is crucial for accurate VT substrate identification and guiding catheter ablation procedures.
▪ Late enhancement (LE) CCT has emerged as a promising alternative to CMR, offering superior spatial resolution, reduced susceptibility to device-related artifacts, and shorter acquisition times. It is particularly useful in scenarios where CMR is limited, such as in patients with implantable devices.
▪ There is a need for standardized protocols for LE-CCT acquisition and analysis, as well as improved workflows for coregistering imaging data with EAM systems. This standardization is essential to enhance the accuracy and efficiency of VT substrate identification and catheter ablation.
▪ The cost-effectiveness of preprocedural imaging remains an important consideration. Although advanced imaging techniques provide significant benefits, their high costs necessitate careful patient selection to optimize the use of these modalities. Further investigation is required to determine the most cost-effective strategies for implementing image-guided interventions in clinical practice.

## Background

Ventricular tachycardia (VT) is a major cause of sudden cardiac death (SCD).^1^ Structural abnormalities generally underlie the development of VT, disrupting the functional electrical activation of the ventricular myocardium.^2^ Scar-related reentry is the most common underlying mechanism of this disturbed electrical activation. The reentrant circuit is generally formed by areas of dense scar surrounded by regions of slow conduction (border zone [BZ] and conduction channels [CCs]). These circuits are connected to healthy myocardium and create the circumstances for substrate reentry, namely, delayed and fractionated electrical activity.^3^

Radiofrequency (RF) catheter ablation is an established treatment method for VT and has been proven to reduce implantable cardioverter-defibrillator therapy and VT burden.^4^^,^^5^ Electroanatomic (voltage) mapping (EAM) is used to identify and target the arrhythmogenic substrate for ablation.^6^ However, there are important constraints to EAM-based substrate assessment, for example, nontolerated VTs and the depiction of 3-dimensional (3D) substrate as a 2-dimensional (2D) image.^7^ Even though there have been significant improvements in both mapping and ablating techniques,^8^ reported postablation recurrence rates remain around 25%–50%.^9^ This warrants an improvement of the existing substrate characterization strategies to improve both acute and long-term ablation success rates.

The 2022 European Society of Cardiology guidelines for the management of VT emphasize the utility and importance of imaging for substrate characterization.^10^ Preprocedural imaging techniques can be used to assess anatomy and tissue characteristics, which subsequently can be used to create 3D cardiac models for guidance of catheter ablation procedures.^11^ Previous studies have reported that cardiac magnetic resonance (CMR) and cardiac computed tomography (CCT) can be applied to identify the target for ablation.^12^, ^13^, ^14^, ^15^, ^16^, ^17^, ^18^, ^19^, ^20^, ^21^, ^22^, ^23^, ^24^, ^25^, ^26^, ^27^, ^28^, ^29^, ^30^, ^31^, ^32^, ^33^ CMR has been used for almost 25 years to characterize myocardial tissue. An important milestone was findings that demonstrated the ability of CMR to further differentiate between scar tissue and BZ of the arrhythmogenic substrate.^12^^,^^13^ More recently, substrate detection using CCT has also shown the promise to accurately identify critical VT isthmuses in postinfarct VT.^26^ However, despite having proven value in assessing VT substrate, there is still an ongoing debate regarding a standardized imaging and postprocessing workflow.

The aim of this study was to review current available literature on image-based VT substrate detection. To this extent, this study investigated the evidence of identifying VT substrate using CMR and CCT compared to an invasive approach. Additionally, we aimed to consolidate these findings and make a concise and simplified recommendation toward standardizing the preprocedural workup in patients with VT.

## Substrate characterization using CMR

CMR is the cornerstone for noninvasive identification of VT substrate,^11^ guiding catheter ablation procedures.^20^ and providing insights into the potential risk of VT recurrences.^3^ Late gadolinium enhancement (LGE)-CMR can reliably identify scar and BZ^12^^,^^14^ and offers crucial insights into the location, mass, and transmurality of substrate in both ischemic cardiomyopathy (ICM) and nonischemic cardiomyopathy (NICM).^34^ In addition, multiple novel metrics have been proposed to further improve substrate characterization, such as entropy,^35^ wall thickness (WT),^14^ and intramyocardial fat.^36^ However, the application of these metrics in the clinical setting remains constrained, primarily because of the absence of consensus on image acquisition and postprocessing strategies (eg, thresholds and cutoff values).

### Image acquisition

Traditionally, 2D sequences in LGE-CMR have been used with a standard in-plane resolution of 1.4–1.8 mm and a slice thickness of 6–8 mm.^37^ However, a recent study indicates the inadequacy of this resolution for precise assessment of VT substrate.^38^ Using a postinfarct porcine model by Pashakhanloo et al^38^ revealed critical CCs within surviving tissue structures with dimensions beyond the capabilities of 2D imaging, necessitating higher spatial resolution.

Recent advancements have facilitated high-resolution 3D acquisitions with isotropic resolutions as low as 1.25 mm.^39^ This increase in spatial resolution has substantially enhanced the delineation and visualization of scar composition and architecture, thereby improving the identification of CCs critical for VT substrate *(critical isthmus).* However, despite these developments, widespread clinical adoption of 3D LGE-CMR remains limited, primarily because of long image acquisition times (at least 1.5× longer)^15^ and higher susceptibility to noise. In addition, there are challenges in contrast differentiation because of similar contrast washout phases of fibrosis and healthy tissues, which recently have been resolved by applying dynamic adjustment of inversion time (TI) during 3D LGE-CMR acquisition.^40^ Based on the analyzed evidence, a high-resolution 3D-LGE imaging strategy seems to be the most appropriate approach for comprehensive VT substrate assessment. In case 2D is required (eg, patient-related constraints), decreases in sensitivity and specificity are due to the limited tissue coverage and spatial resolution.

### Postprocessing

Following acquisition of LGE-CMR, the subsequent step in the workflow involves the quantification of abnormal myocardium. It has been established that VT substrate primarily resides within the BZ, making it essential to move beyond visual grading of LGE imaging for an accurate assessment of VT substrate.^41^ Various methods have been proposed and applied for post-processing LGE-CMR images, including manual quantification, semi-automatic thresholding, and fully automated segmentation. Initially, manual quantification involved segmenting regions of interest based on signal intensity.^42^ However, this approach is time-consuming and error-prone, and lacks reproducibility, whereas fixed-thresholding methods offer a semiautomatic alternative.^43^

Recent years have seen the advent of commercial platforms such as MUSIC/InHeart (IHU LIRYC Bordeaux and Inria Sophia, France) and ADAS 3D (Adas3D Medical S.L., Barcelona, Spain), which offer advanced capabilities for processing CMR and CCT data, automatically characterizing scar differences and differentiating between core, BZ, and CCs.^16^^,^^44^ These platforms use differences in signal intensity and apply standard deviation (*n*-SD) or full-width half-maximum (FWHM) thresholding methods to segment abnormal myocardium.^45^ FWHM defines the threshold as half of the maximum signal intensity within the scar. The *n*-SD method defines the fixed threshold as the sum of the mean and 2 or *n* SDs of signal intensities in a reference region.

The availability of advanced postprocessing tools has increased the reproducibility and feasibility of creating 3D substrate models. However, a direct comparison between the software platforms has yet to be performed. Furthermore, both thresholding methods suffer from inaccuracies. For example, the *n*-SD method is severely user-dependent,^45^ with FWHM demonstrating greater reproducibility in scar segmentation and less prone to over- or underestimation.^46^ A major limitation of FWHM is the inaccuracy in segmenting less bright or fragmented scar cores and inhomogeneous gray scars.^42^ In addition, FWHM, like *n*-SD, is user-dependent and requires manual input. Fixed-thresholding methods may struggle to adapt to variations in scar architecture, such as the presence of diffuse fibrosis, potentially leading to overestimation of substrate.^47^ In addition to fixed-thresholding methods, more recent investigations have leveraged deep learning algorithms for automated segmentation, although their efficacy has not been extensively validated, and they remain less accessible because of their bespoke nature.^45^

In essence, the variations in scar characteristics may introduce inaccuracies in semiautomatic thresholding methods. Given the current evidence, using (semi-)automated software platforms for quantification of abnormal myocardium is recommended for postprocessing of LGE-CMR imaging. A commonly used cutoff value for FWHM is around 50% (40%–60%), and 2–6 SDs typically are used to classify abnormalities.^46^^,^^48^ Specifically, 2–3 SDs are recommended for identification of VT substrate because of an increased specificity.

### Correlation of CMR with EA(V)M

#### Structural assessment

**Figure 1:**
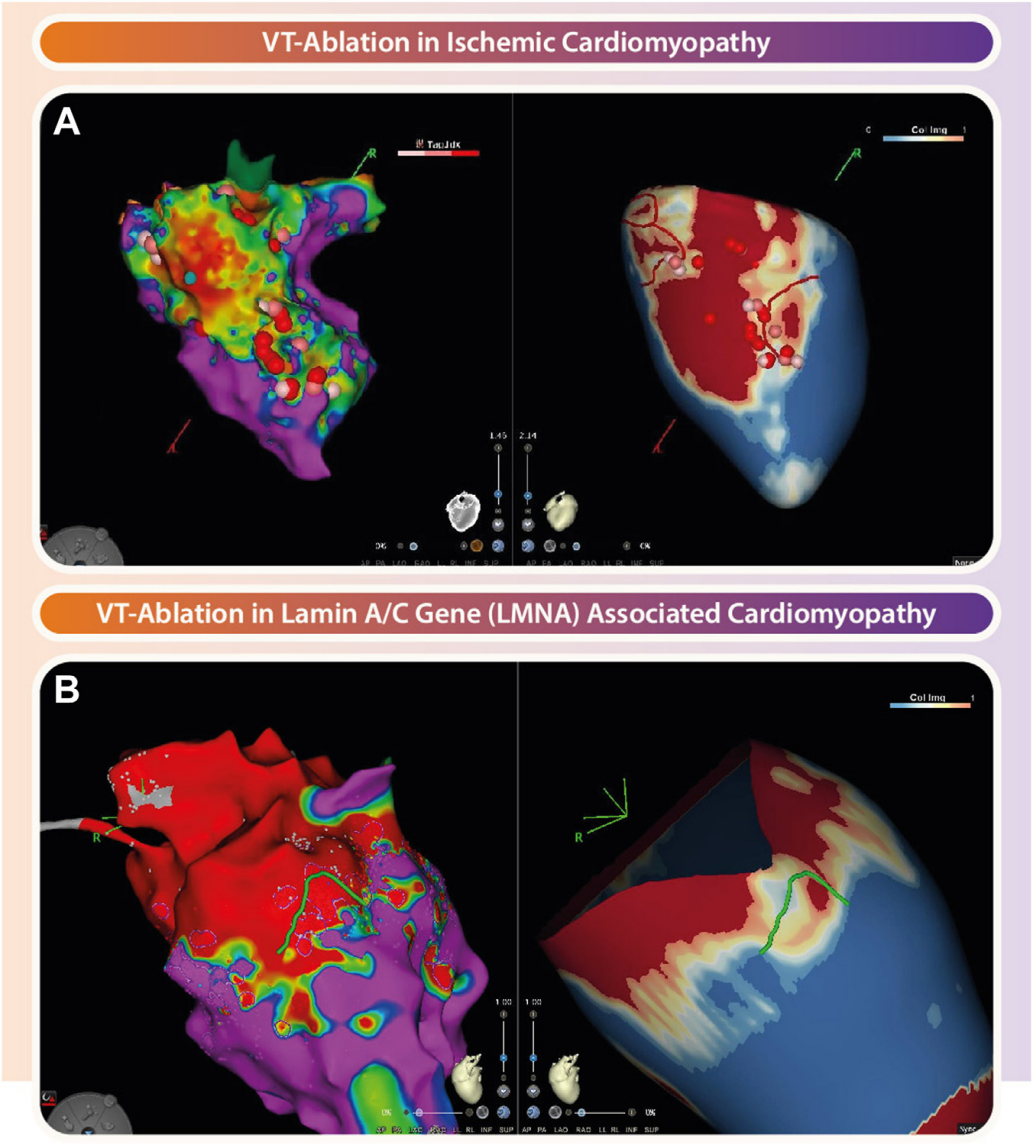
Role of magnetic resonance imaging–derived scarmaps in guiding ventricular tachycardia (VT) ablation. **A:** Electroanatomic map (EAM) **(left)** and cardiac magnetic resonance–derived scarmap **(right)** of a patient with ischemic cardiomyopathy where ablation of the conduction channel (CC) rendered the VT noninducible. **B:** Comparable overview with EAM **(left)** and scarmap **(right)** of a patient with nonischemic cardiomyopathy where activation mapping was not feasible due to hemodynamic instability during VT. Fourt different VT foci were identified using targeted mapping in regions with border zone and CCs. The CC, which traversed through a large midwall septal substrate, was ablated, resulting in noninducibility of the clinical VT.

Currently, VT ablation techniques rely on EAM systems to assess structural and functional characteristics of arrhythmic substrates. Notably, studies have extensively compared EAM with CMR,^15^^,^^17^ Z(Figure 1) and have demonstrated the feasibility and efficacy of integrating these modalities to guide catheter ablation procedures, particularly in scar-related VT.^18^

Integration between EAM and CMR is achieved by exporting postprocessed 3D scarmaps, which subsequently are imported into procedural navigation systems such as CARTO (Biosense Webster, Inc., Diamond Bar, CA) or EnSite NavX (St. Jude Medical, Inc., St. Paul, MN), and registered with EAM using distinguishable landmarks.^44^ Anatomic landmarks, such as the pulmonary artery, left main coronary artery, right ventricle, or left ventricular apex, serve as recognizable and reliable fiducial points.^13^^,^^19^^,^^20^ An important aspect of image integration is the potential rotation of cardiac compartments, which can be overcome using aortic landmarks.^13^

The recommended workflow for image integration is as follows:

1. Acquire high-resolution 3D LGE-CMR images with isotropic resolutions of at least 1.25 mm, ensuring coverage of the entire ventricular myocardium
2. Use advanced postprocessing software platforms such as MUSIC/InHeart or ADAS 3D to segment and generate detailed 3D scarmaps
3. Select and annotate the following anatomic landmarks in both imaging and EAM datasets, preferably all, but at least a and b:
  a. Bifurcation of pulmonary artery
  b. Inferior and superior caval vein
  c. Aortic root
  d. Aortic arch
4. Import the scarmap into the EAM system and perform point-by-point matching to align the 3D model with the patient's cardiac anatomy
5. Validate the coregistration by comparing EAM voltage maps with image-derived map, adjusting as needed to ensure precise alignment

The workflow for integrating CT with EAM follows a similar process, with adjustments for imaging modality differences, ensuring high-resolution LE CT scans and the same steps for segmentation, landmark identification, coregistration, and validation are followed.

Early studies, including the pioneering work by Wijnmaalen et al,^19^ underscored the importance of real-time integration of LGE-CMR– derived scarmaps and voltage maps in postinfarction VT patients. This study emphasized the significance of integrating CMR and EAM (bipolar voltage cutoff <1.5 mV), as EAM alone was unable to identify the complexity of scar in case of nontransmural and heterogeneous scar, particularly in patients with subendocardial scar with <75% transmurality. Following this landmark study, various other institutes reported successful image integration.^13^^,^^16^^,^^22^^,^^44^^,^^49^ Ongoing advances in postprocessing software have enabled the identification of heterogeneous tissue channels (HTCs) that can be integrated in EAM systems and used to perform targeted VT ablation.^13^ More recently, studies have added an extra dimension to HTCs by investigating specific arrhythmogenic characteristics, such as mass and protectedness, in an attempt to further improve the definition of VT circuit arrhythmogenicity.^21^ HTCs related to a VT isthmus are longer, and have higher mass, higher transmurality, and, in particular, a higher degree of protectedness (fully protected: >40% core scar; partially protected: 15%–40% core scar) than those that were not related to a “clinical” VT isthmus.

Despite these promising advances, challenges such as potential mismatches between EAM and CMR data due to EAM inaccuracies, including catheter-related limitations such as interelectrode spacing^50^ and inadequate tissue contact,^51^ still persist. In addition, inaccuracies associated with image quality (breathing, inadequate nulling) and the use of fixed cutoff thresholds are potential sources of discrepancies. Furthermore, EAM low-voltage areas are not necessarily a reflection of the true arrhythmogenic substrate as a voltage-based approach is unable to account for functional arrhythmic aspects.^52^

#### Functional assessment

Various functional mapping strategies have emerged and undergone clinical validation, for example, deceleration zones (DZs) and decrement evoked potential mapping. However, literature regarding the correlation of functional substrate assessed using invasive electrophysiological study with substrate quantified using CMR is limited.

A recent study by Vázquez-Calvo et al^17^ revealed a strong correlation between DZs derived by isochronal late activation mapping and the CCs identified by CMR scarmaps. These DZs appear in different areas when remapping, however, consistently align with the CMR channels, thus highlighting the capability of CMR to identify slow conducting areas. Nonetheless, isochronal late activation mapping has limitations, including its time-consuming nature, the assumption that slow conduction zones correspond solely to VT substrate, and its dependency on activation sites,^53^ leading to its infrequent use in clinical settings. In contrast, decrement evoked potential mapping presents an alternative approach to identify VT substrate by analyzing ventricular electrograms exhibiting decremental conduction.^54^^,^^55^ These regions often colocalize with areas initiating VT, offering promise for substrate identification without the necessity for VT induction.

In conclusion, notwithstanding potential challenges such as registration errors and time-intensive workflows, the integration of CMR and EAM remains invaluable for guiding procedural interventions in scar-related VT.^56^ Although CMR alone has been proven effective in identifying scar-related VT substrate in ICM patients, the complexity of VT substrate in NICM necessitates the integration of CMR with EAM data to comprehensively assess both structural and functional VT substrate, thereby guiding the ablation procedure effectively.^56^ It is important to create a standardized image integration workflow, consisting of postprocessing and predefined landmarks, to optimize outcomes in clinical practice.^22^

### Clinical application of CMR in VT ablation

CMR enables differentiation between ischemic scar (well-defined regions of LGE) and scar in NICM, often associated with a more heterogeneous pattern of scarring, which can be midmyocardial or epicardial. This by itself is useful to tailor the ablation approach, that is, endocardial vs epicardial vs endocardial and epicardial combined. Visualization of newly identified or modified VT circuits through LGE-CMR has been reported to facilitate more effective ablation of recurrent VT after an initial procedure.^24^ In addition, image integration has been linked to other procedural benefits, such as significantly reduced procedural time, less RF application, and lower fluoroscopy times.^20^^,^^22^ Finally, image-guided substrate identification also improves selection of the ablation approach where LGE-CMR has proven utility in accurately guiding epicardial ablation in patients with epicardial-only or predominantly epicardial VT substrates, leading to higher acute ablation success (66.7% vs 22.2%).^57^ A particularly interesting study was performed in patients with hypertrophic cardiomyopathy, in which it was demonstrated that scar architecture significantly influences the inducibility and electrophysiological traits of ventricular arrhythmia in hypertrophic cardiomyopathy. Scar mass increased was shown to be predictive for inducibility of nonsustained and sustained VT and could be used to select patients for catheter ablation.^58^

Studies exploring the utility of CMR in guiding VT ablation procedures have indicated that CMR-guided VT ablation is linked with superior acute ablation success rates, noninducibility rates, and VT recurrence-free survival following substrate ablation.^20^^,^^22^, ^23^, ^24^ However, amid these promising findings, some studies have presented contrasting results. For example, recent research has suggested that although CMR-guided ablation exhibits lower recurrence rates, it does not significantly alter additional acute and long-term outcomes (survival) compared to a control group without CMR guidance.^49^

**Figure 2:**
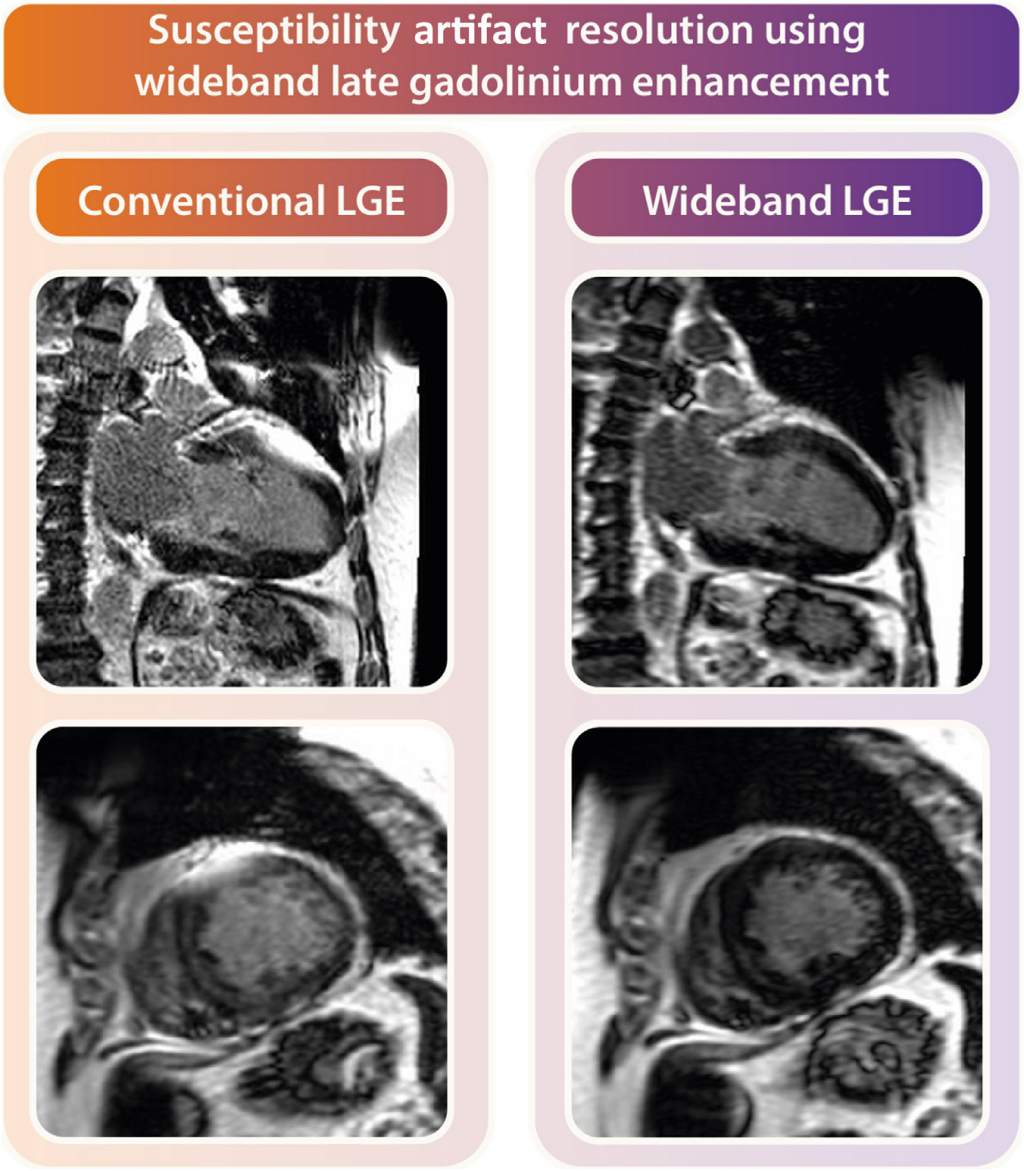
Clinical utility of wideband late gadolinium enhancement (LGE) for artifact suppression. **Left:** Conventional long- and short-axis LGE images with a large susceptibility artifact from the can of an implantable cardioverter-defibrillator on the left ventricular anterior wall. **Right:** Wide-band LGE images in the same patient with resolution of the artifact providing diagnostic quality images.

Overall, there still remains a big gap in the literature, as image-guided ablation studies have only been performed in a limited number of NICM patients. In addition, the etiology of the underlying cardiomyopathy (eg, arrhythmogenic right ventricular cardiomyopathy or myocarditis) presents unique challenges in generalizing the results obtained to date. Furthermore, it must be noted that although CMR guidance is greatly beneficial, the diagnostic yield of CMR often is significantly compromised in clinical settings where patients have implanted cardiac devices.^59^ Despite various efforts to address this challenge using wideband sequences^25^ (Figure 2), device-related artifacts as a result of patient-specific limitations (eg, low position of the device can) remain reality. Because CMR is not applicable in all cases, which prompts exploration of alternative imaging modalities such as CCT for visualization of arrhythmic substrate.

## Shift toward CCT

**Figure 3:**
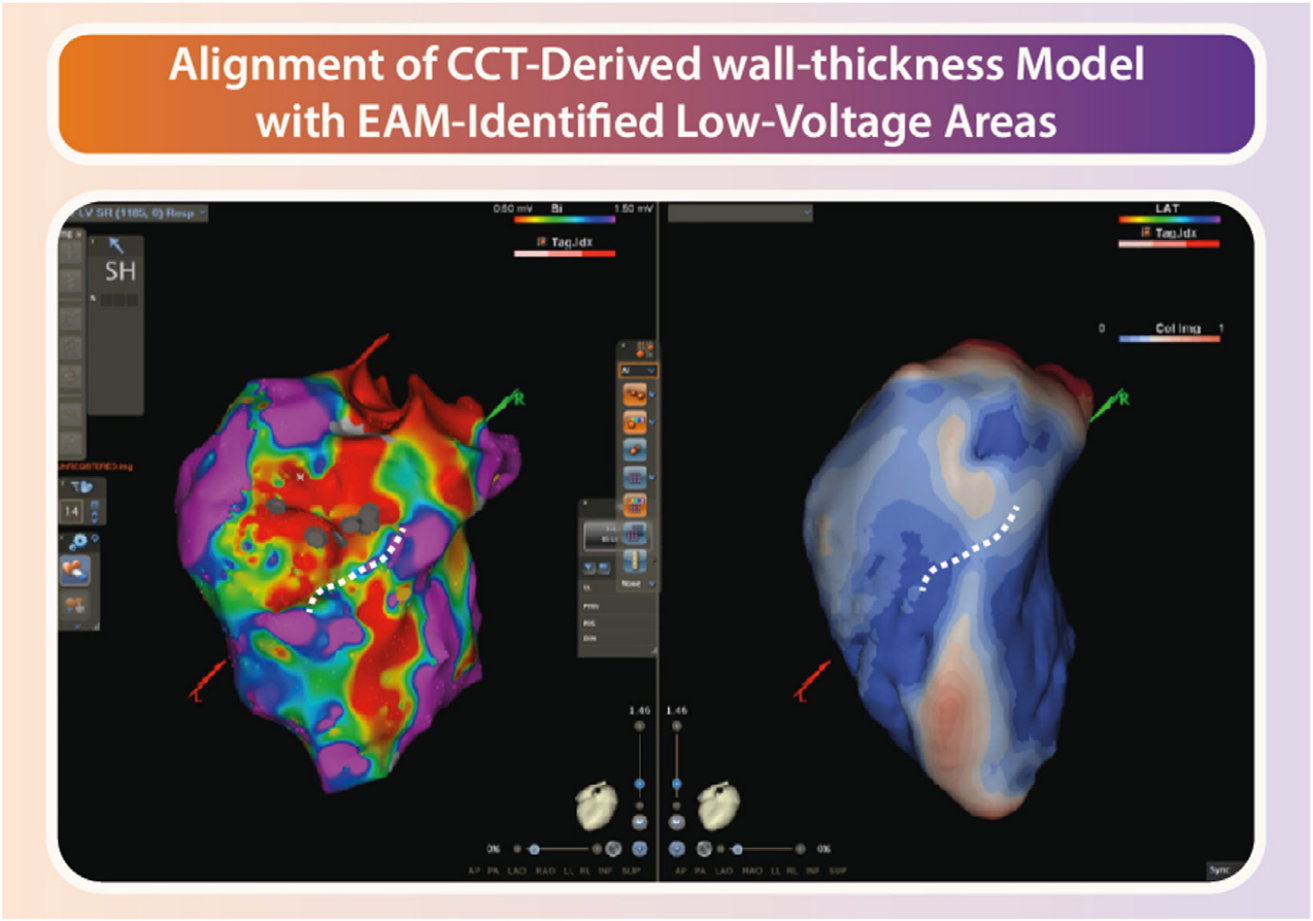
Cardiac computer tomography (CCT)-derived wall thickness. Wall thickness analysis from computer tomographic imaging in a patient with ischemic cardiomyopathy demonstrating a conduction channel (*blue area;* normal wall thickness) surrounded by wall thinning *(red areas).* EAM = electroanatomic mapping.

In recent years, a steady increase can be observed in the application of CCT for guidance of VT ablation procedures.^26^, ^27^, ^28^, ^29^^,^^60^ A retrospective study analyzing 108 patients compared VT ablation aided by late-enhancement CT analysis and segmentation with conventional methods such as substrate and activation mapping.^29^ Results showed a lower VT recurrence rate in the image-guided group (27% vs 42%; *P* <.06); in particular, patients with ICMs benefited significantly and had higher success rates (*P* = .05). Similarly, in another study of 42 consecutive patients referred for VT ablation, preprocedural CT scan identified scar in 39 patients and demonstrated a good segmental concordance between CT and EAM (κ = 0.536).^26^ CT showed good sensitivity (76%), specificity (86%), and very high negative predictive value (NPV) (95%) in identifying low-voltage segments. In addition, late potentials and RF ablation points aligned with CT-identified scarred segments in 79% and 81% of cases, respectively. Finally, a point-by-point comparison showed a strong correlation between the average scar area detected by CT and bipolar mapping (R = 0.78; *P* <.0001), with 70% and 84% of low-amplitude bipolar points within 5 and 10 mm of CT-segmented scars, respectively. More recently, Borrego et al^61^ conducted multidetector CT on patients undergoing VT ablation and postprocessed the images using myocardial thickness analysis software, creating a 3D color map identifying scar dense/border zones (DZ/BZ) and CCs. There was a strong anatomic correlation between DZ, BZ, and CC detected on multidetector CT images and the pathologic voltage areas and late electrograms on the omnipolar electroanatomic map. In ICM, the CC identified on CT coincided with the diastolic corridor of clinical VT, demonstrating the accuracy of the imaging technique in localizing critical structures. An example of this is shown in Figure 3.

Overall, the integration of CCT in VT ablation procedures provides a detailed anatomic roadmap that enhances the precision and effectiveness of the ablation, leading to improved outcomes and reduced complications. These studies collectively underscore the importance of CCT in optimizing VT ablation strategies by providing critical insights into the heart's arrhythmogenic substrates. This can be attributed to several advances, including reduced acquisition time, lower radiation exposure, and enhanced temporal resolution, all of which have broadened the scope of CT in cardiac interventions. Moreover, CCT offers the distinct advantage of superior spatial resolution and reduced susceptibility to device-related artifacts, which has led to preliminary investigations into the potential of late enhancement (LE) CCT to identify scar tissue^28^^,^^62^^,^^63^ and VT substrate.^26^^,^^29^ However, because of its recent introduction, the current landscape lacks a standardized approach for the acquisition and analysis of LE-CCT.

### Image acquisition

Given the absence of an “established” protocol, the acquisition approach of LE-CCT, in contrast to LGE-CMR, differs tremendously between previously conducted studies. Supplemental Table 1 highlights the discrepancies observed in the administration of contrast agents and CT acquisition parameters in both clinical and preclinical investigations aimed at identifying scar or VT substrate.

#### Contrast

Late iodine enhancement CT has demonstrated efficacy in detecting postinfarction scar areas akin to that of LGE-CMR.^64^^,^^65^ Variations in the choice, dosage, and timing of contrast agent administration are evident (Supplemental Table 1). There is a large variation in timing delay after contrast administration (range 5–15 minutes). The majority of investigations have shown that a delay time of 5–8 minutes is adequate for achieving optimal image quality.^28^^,^^62^^,^^65^ Specifically, in the assessment of myocardial infarction, a delay phase of 6–8 minutes is most commonly used and therefore recommended.^66^

Beyond contrast dosage and timing, image acquisition settings significantly influence the resulting image quality. Adjustments in parameters such as tube voltage/potential (kV) and tube current (mAs) play a crucial role in optimizing myocardial contrast and minimizing radiation exposure.^26^ A lower tube voltage during delayed acquisition has been advocated to maintain image quality while reducing radiation dose.^67^ Modulating tube voltage between 80 and 100 kV based on patient weight ensures adequate image quality.^63^ A tube voltage of 80 kV is generally recommended for VT substrate detection. Another important variable is tube current, which can impact acquisition speed. Higher tube currents (310–550 mAs) lead to faster acquisition with maintained enhanced contrast at a lower tube voltage (80–100 kV) but subsequently increased radiation exposure. Nonetheless, a low tube current (160 mAs) has shown moderate accuracy in the detection of myocardial scar.^62^ Therefore, the tube current is advised to minimize the radiation while ensuring image quality at 310 mAs.

Recent advancements in imaging techniques propose the use of multispectral CT, which involves simultaneous acquisition of images at multiple energy levels. Research indicates that dual-energy CT can assess myocardial scar and LE compared to CMR.^68^ However, certain studies, notably animal research, have shown that single-energy LE-CCT outperformed dual-energy CT in scar detection compared to EAM and pathologic tissue assessment.^69^ The application of novel multispectral CT is still in its early stages and warrants further exploration. Thus, based on current evidence, single-energy LE-CCT is recommended for assessing VT substrate.

### Postprocessing and correlation with EA(V)M

Following the acquisition of LE-CCT images, the subsequent phase involves postprocessing, which shares a similar workflow to that of CMR. This encompasses manual or automatic segmentation of regions of interest. As previously outlined, (semi-)automated segmentation techniques, including machine learning algorithms and specialized software tools, are viable options for CT postprocessing. Commercially available software tools leverage previously mentioned segmentation techniques for (semi-)automatic postprocessing of CCT data, most often embedded in software platforms such as IntelliSpace Portal or InHeart/Music.^29^^,^^63^

Limited evidence is available on how these CCT-derived substrate models correlate with EAVM data. A study by Esposito et al^26^ revealed a significant agreement between scar areas identified by CCT and low-voltage areas indicated by bipolar voltages in EAM (Figure 3). Additional studies have shown that 3D scar and BZ data correlate well with low-voltage areas in EAM.^27^^,^^28^ Furthermore, results have demonstrated the accuracy of merging CCT-derived scarmaps with EAM to identify myocardial abnormalities, particularly in VT patients with LGE-CMR contraindication.

### Clinical application of CCT in VT ablation

Previous research has demonstrated the efficacy of CCT in accurately identifying critical regions, such as wall thinning,^60^^,^^70^ myocardial calcifications,^27^ and fat tissue,^30^ all of which have been linked to ventricular arrhythmias. One of the first studies investigating WT included 13 consecutive postinfarction patients who underwent CCT before VT ablation.^59^ A significant correlation was found between WT <5-mm areas and endocardial low voltage (R = 0.82; *P* = .001), but not in the epicardium. The WT <5-mm area was smaller than the endocardial low-voltage area (54 ^2^ vs 71 cm^2^; *P* = .001). Of the 13,060 electrograms reviewed, 538 local abnormal ventricular activities were detected, mostly located within WT <5 mm (87%) or at its border. Very late local abnormal ventricular activities (>100 ms after QRS) were predominantly found in WT <3-mm areas (93%). A recent study in 34 patients undergoing VT ablation demonstrated 94.1% of primary DZs located in areas of wall thinning in ICM patients compared to 20% in NICM patients overall, but 50% if any wall thinning was present. Fifty-nine percent of DZs in ICM patients and 56% of DZs in NICM patients were located near wall thinning centers. The positive predictive value (PPV) of wall thinning centers in localizing DZs was 22.5% in ICM patients and 37.8% in NICM patients.^71^ Incremental benefit in prediction of arrhythmogenic substrate has been demonstrated using lipomatous metaplasia. A recent study of 19 patients (14 ischemic, 5 nonischemic) demonstrated that in ischemic patients, lipomatous metaplasia had 70% sensitivity, 89% specificity, 73% PPV, and 88% NPV in predicting conducting channels compared to CT channels (10% sensitivity, 98% specificity, 70% PPV, 72% NPV) and wall thinning (61% sensitivity, 82% specificity, 58% PPV, 84% NPV). For nonischemic patients, lipomatous metaplasia had 69% sensitivity, 81% specificity, 39% PPV, and 94% NPV for predicting conducting channels compared to CT channels (15% sensitivity, 100% specificity, 100% PPV, 87% NPV) and wall thinning (46% sensitivity, 82% specificity, 32% PPV, 89% NPV).^72^

Although the utility of LE-CCT in assessing VT substrates has not yet been extensively explored, preliminary work is already suggesting potential benefits in CT guidance for ablation procedures.^26^ Notably, VT ablation using LE-CCT has shown promise in achieving acute procedural success and reducing VT recurrence rates (27%) compared to conventional (electrogram-based) mapping and ablation approaches (42%).^29^ In contrast, findings from Bettencourt et al^62^ indicate that LE-CCT exhibits only moderate accuracy in detecting ischemic scar tissue compared to CMR. LE-CCT detected ischemic scar in 52.9% of the patients identified by CMR, with reported sensitivity of only 53% but specificity of 98%. Nonetheless, research suggests that in VT patients referred for ablation and with CMR contraindication, CCT can accurately detect substrate.^28^

Although the validation of myocardial assessment using LE-CCT remains less extensive compared to LGE-CMR, the widespread availability and increasing evidence demonstrating clinical value of LE-CT make it a valuable tool in scenarios where CMR may be limited in detecting substrate, for example, in the presence of implantable cardioverter-defibrillator artifacts or general contraindications for undergoing CMR.

## Imaging of cardiac-adjacent structures

Preprocedural imaging of cardiac-adjacent structures plays an important role in the planning and execution of VT ablation procedures. Accurate imaging of coronary arteries, the phrenic nerve (PN), and other adjacent structures can significantly enhance the safety and efficacy of these interventions.

Advanced imaging techniques, such as CMR and CCT, have demonstrated high accuracy in visualizing coronary arteries and the PN. CMR, particularly with LGE, provides detailed insights into myocardial tissue characteristics and adjacent structures. CCT, with its superior spatial resolution, offers precise anatomic details, making it possible to visualize coronary arteries, myocardial bridges, and other structures with high accuracy.

### Coronary arteries

Preprocedural imaging has the potential to reduce or even eliminate the need for coronary angiography at the time of epicardial ablation. Studies have shown that CCT can provide sufficient anatomic detail to identify coronary arteries and assess their patency.^73^ The accurate imaging of coronary arteries is critical, especially during epicardial ablation procedures in which the risk of damaging these vessels is high. CCT is particularly effective in mapping the course of the arteries and helps in planning ablation paths that avoid coronary arteries, thereby reducing the risk of vascular complications. This noninvasive approach can streamline the procedural workflow, reduce patient exposure to contrast agents and radiation, and decrease the overall procedural time.

### PN

Imaging of the PN is essential to prevent PN injury during epicardial ablation. CCT has shown high efficacy in detecting the PN and assessing its anatomic variability. In a study involving 95 consecutive patients with VT, CCT detected the PN in 81 patients (85%).^74^ Epicardial mapping was conducted in 49 patients, using real-time CCT integration to display coronary artery and PN locations in 3D mapping systems. It was demonstrated that epicardial VT substrate is frequently located near coronary arteries and the PN, and integration of high-resolution imaging is highly beneficial in reducing the risks of injury to these structures during epicardial VT ablation.

Overall, numerous studies have validated the use of CMR and CCT for imaging cardiac-adjacent structures. However, additional research is essential to standardize imaging protocols and confirm their efficacy across diverse patient populations. Comparative studies evaluating the outcomes of ablation procedures guided by preprocedural imaging vs traditional approaches would be particularly valuable in establishing standardized preprocedural imaging practices.

## Discussion

Both CMR and CCT offer valuable insights into the structural and functional abnormalities underlying VT. CMR, particularly with LGE, has established itself as a cornerstone in noninvasive VT substrate identification, providing crucial information on scar tissue, BZs, and conducting channels. However, challenges such as manual postprocessing and limited resolution in traditional 2D imaging highlight the need for advancements such as high-resolution 3D acquisitions and automated segmentation techniques. Integration of CMR with EAM systems presents a robust approach for guiding catheter ablation procedures, allowing for comprehensive assessment of scar-related VT substrates. Although challenges such as registration errors persist, the benefits of integrating CMR and EAM is an ongoing field of research, especially in distinguishing scar core from BZs and identifying HTCs crucial for targeted ablation.

In contrast, CT imaging, particularly with LE, has emerged as a promising alternative, offering advantages such as superior spatial resolution, reduced susceptibility to device-related artifacts, and shorter acquisition times. Despite lacking a standardized approach for LE-CCT acquisition and analysis, recent studies have demonstrated the potential of CCT in accurately identifying scar tissue and guiding VT ablation procedures. Integration of CCT with EAM data provides valuable procedural guidance, although discrepancies between CCT and EAM data necessitate careful consideration. Nonetheless, the clinical application of CCT in VT ablation has shown promising results, particularly in scenarios where CMR may be limited, such as in the presence of implantable devices.

The cost-effectiveness of preprocedural imaging is a critical consideration often overlooked in discussions about technological advancements in VT ablation. Although advanced imaging techniques such as LE-CCT and LGE-CMR offer significant benefits, they come with substantial costs. Additionally, image processing services provided by companies can add to the overall expense. Given the constraints of health care economics, it may not be feasible to offer these advanced imaging techniques to all patients in all practices. Therefore, it is essential to identify high-yield patients who would benefit the most from preprocedural imaging to mitigate costs effectively. For example, preprocedural imaging may be more useful in patients with NICM compared to those with ischemic/postinfarct VT, or in cases where 12-lead electrocardiography of the VT is not available, suggesting a more ambiguous substrate location. Prioritizing such patients can enhance the cost-effectiveness of these advanced imaging modalities.

**Figure 4:**
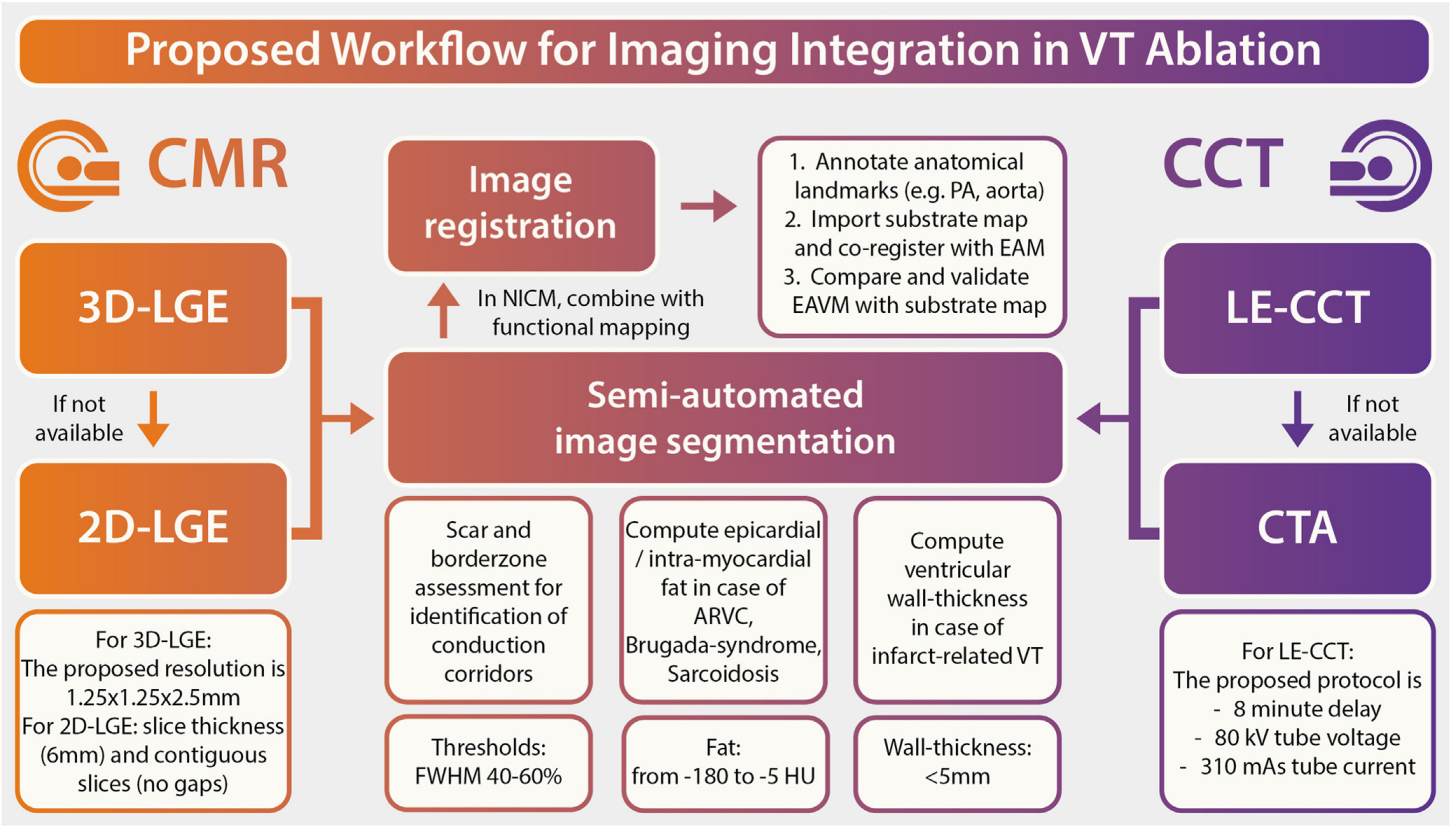
Recommendations for a standardized image acquisition and analysis workflow before ventricular tachycardia (VT) ablation. A concise overview of recommended imaging-strategies for optimizing preprocedural imaging in patients undergoing VT ablation. If available, 3-dimensional late gadolinium enhanced (3D-LGE) imaging should be preferred over 2-dimensional (2D)-LGE. Scarmaps, processed using commercially available software, can significantly facilitate procedural guidance. If no cardiac magnetic resonance (CMR) imaging is available, late enhancement cardiac computed tomography (LE-CCT) or CT angiography (CTA) should be acquired before VT ablation. LE analysis or wall thickness in conjunction with intramyocardial fat can be used to generate comparable scarmaps. Integration of CMR or CT-derived scarmaps with the electroanatomical system should be performed by using standardized landmarks such as the pulmonary artery (PA) or aorta. These recommendations aim to enhance the accuracy and efficiency of substrate identification, ultimately improving the precision and efficacy of VT ablation procedures. ARVC = arrhythmogenic right ventricular cardiomyopathy; EAVM = electroanatomic voltage mapping; FWHM = full-width half-maximum.

Overall, although each modality presents unique advantages and challenges, their integration with EAM systems can significantly improve the precision and efficacy of VT ablation procedures. To achieve this goal, it is imperative to establish a comprehensive image acquisition and analysis workflow that optimally utilizes the strengths of both CMR and CT imaging modalities. Based on the extensive review of literature, the following recommendations are suggested (Figure 4). Three-dimensional LGE imaging is recommended. In cases where cardiac magnetic resonance imaging is not feasible, CT-LE or CTA should be performed before VT ablation. Analysis of LE or WT, in conjunction with intramyocardial fat, can generate scarmaps from both CMR as well as CCT, which might be helpful in preprocedural substrate assessment. Integration of these scarmaps with the electroanatomic system should be standardized using landmarks such as the pulmonary artery or aorta. These recommendations are designed to enhance the accuracy and efficiency of substrate identification, thereby improving the precision and efficacy of VT ablation procedures.

## Future perspectives

The future of imaging in VT is poised for significant advancements, driven by emerging technologies and innovative approaches. These advancements promise to enhance the precision, efficacy, and safety of VT ablation procedures, ultimately improving patient outcomes.

### Photon-counting CT

One of the most promising emerging technologies is photon-counting CT. Unlike conventional CT, photon-counting CT detectors measure the energy of individual x-ray photons, which allows for superior tissue characterization while maintaining high spatial resolution. This technology significantly reduces noise, including artifacts from cardiac devices such as implantable cardioverter-defibrillators, which traditionally have posed challenges in imaging. Photon-counting CT can distinguish between different tissue types with greater accuracy, providing detailed insights into myocardial structure and scar tissue. This enhanced imaging capability can lead to more precise identification and targeting of arrhythmic substrates, improving the efficacy of VT ablation procedures.

### Artificial intelligence

Artificial intelligence and machine learning are set to play a pivotal role in the future of cardiac imaging. These technologies can automate and enhance image analysis, offering rapid and accurate segmentation of myocardial scars and identification of VT substrates. Artificial intelligence–driven algorithms can integrate multimodal imaging data, including CT, MRI, and electroanatomic maps, to create comprehensive 3D models of the heart. These models can aid in planning and executing precise ablation strategies, reducing procedural times and improving success rates. Furthermore, artificial intelligence can continuously learn and adapt from clinical outcomes, refining its algorithms to enhance future patient care.

## Conclusion

CMR and CCT both offer valuable insights into VT substrate characterization, with CMR serving as a cornerstone in noninvasive assessment and CT emerging as a promising alternative. Future directions warrant further standardization of imaging protocols and postprocessing techniques, to advance VT substrate identification and ablation outcomes. Ongoing efforts, refining image protocols and postprocessing, are essential to optimize the integration of CMR and CCT into routine clinical practice.
