# Supplementary material for: Optimizing ventricular tachycardia ablation through imaging-based assessment of arrhythmic substrate: A comprehensive review and roadmap for the future
Source: Heart Rhythm O2. 2024 Jul 5;5(8):561–72. doi: 10.1016/j.hroo.2024.07.001 (PMC11385403; doi:10.1016/j.hroo.2024.07.001)
Supplement: Supplemental Material [file mmc1.docx]

**Supplemental material**

**- Search strategy.** A description of the literature search strategy.

**- Table 1.** Acquisition approaches studies using LE-CCT for the assessment of myocardial scar or VT substrate.

**Search strategy**

Data source and search strategy

A PubMed search was conducted to identify publications focusing on CMR and/or CCT imaging modalities for VT substrate mapping and ablation within the timeframe of 2008 to 2023. The search strategy incorporated the following keywords: Ventricular tachycardia, Catheter ablation, Electroanatomic mapping, Cardiac imaging. The detailed PubMed search is provided in appendix 3. Lastly, the search results were limited to English language papers.

Search MeSH terms

(("tachycardia, ventricular"[MeSH Terms] OR "ventricular arrhythmias*"[Title/Abstract] OR "ventricular tachycardia*"[Title/Abstract] OR "tachycardia*"[Title/Abstract] OR "tachycardias*"[Title/Abstract] OR "vt"[Title/Abstract] OR "ventricular arrhythmia*"[Title/Abstract]) AND ("Catheter Ablation"[MeSH Terms] OR "ablation*"[Title/Abstract] OR "ablations*"[Title/Abstract] OR "radiofrequency ablation*"[Title/Abstract] OR "cardiac ablation*"[Title/Abstract] OR "radioablation*"[Title/Abstract]) AND ("topography, medical"[MeSH Terms] OR "substrate mapping*"[Title/Abstract] OR "mapping*"[Title/Abstract] OR "electroanatomical mapping*"[Title/Abstract] OR "electroanatomic mapping*"[Title/Abstract] OR "arrhythmogenic substrate*"[Title/Abstract] OR "isthmus*"[Title/Abstract] OR "myocardial scar*"[Title/Abstract]) AND ("Magnetic Resonance Imaging"[MeSH Terms] OR "tomography, x ray computed"[MeSH Terms] OR "cardiac magnetic resonance*"[Title/Abstract] OR "magnetic resonance*"[Title/Abstract] OR "cmri*"[Title/Abstract] OR "mri"[Title/Abstract] OR "lge"[Title/Abstract] OR "computed tomography*"[Title/Abstract] OR "contrast enhanced*"[Title/Abstract] OR "ct"[Title/Abstract] OR "computed*"[Title/Abstract] OR "mri ct*"[Title/Abstract] OR "ct mri*"[Title/Abstract] OR "ct and mri*"[Title/Abstract]) AND (humans[Filter]) AND (english[Filter]))

Study selection criteria and data extraction

Studies reporting VT substrate characterization using CMR and/or CCT were included. The outcome measures included the correlation between imaging and EAM, and VT ablation outcomes (acute and long-term). Papers were selected based on their relevance for the research question determined through title and abstract evaluation according to the inclusion and exclusion criteria. Eligible full text papers were then included in the review, excluding animal studies, abstracts-only papers, reviews and meta-analyses. After selection of the qualified papers, the full-text articles were reviewed and analysed. The search strategy identified 194 papers, out of which 111 were excluded based on language, animal studies or reviews and meta-analysis. After assessment of the outcome measures, 58 of the remaining papers were excluded, leading to an inclusion of 25 papers for the review. The study selection process is illustrated in figure 1.

Data synthesis and statistical analysis

The included articles were meticulously analyzed by two reviewers (JCB and PB) to extract the key findings from each study. The objectives, relevant results and relevant conclusions of the papers were documented. Furthermore, the characteristics of the included studies were reported in table 1. The study characteristics include the year of publication, type of cardiomyopathy, cohort size, imaging modality and where applicable the magnet (T). A color-based allocation of the included papers was made based on the imaging modality (CCT, CMR or multimodal). Lastly, figure 2 illustrates the division of the imaging modalities and the different topics within the included papers regarding CMR.

**Table 1.** Acquisition approaches studies using LE-CCT for the assessment of myocardial scar or VT substrate.

| **Study** | **Scanner slices** | **Tube Voltage (kV)** | **Tube Current (mA)** | **Contrast agent (iodine concentration)** | **CA dosage (ml)** | **Waiting Time (minutes)** | **Radiation (mSv)** |
| --- | --- | --- | --- | --- | --- | --- | --- |
| Esposito, et al. 2016 ^32^ | 64-slice | 80kV | - | High iodine (370-400mgI/ml) | 130-140 ml | 10 min | 1.5 ± 1.17mSv  3.9 ± 1.47mSv |
| Bettencourt, et al. 2013 ^66^ | 64-slice | 80kV | 160mA | Iopromide (370mgI/ml) | 80ml | 7 min | 0.5 ± 0.10mSv |
| Conte, et al. 2022 ^34^ | 256-slice | - | - | Iodinated (1.5ml/kg) | 110-133ml | 8 min | 1.1 ​± ​0.3mSv |
| Mahnken, et al. 2005 ^75^ | 16-slice | 80kV | 500mA | Iopromide (370mgI/ml) | 120ml | 15 min | 2.72 ± 0.42mSv |
| Truong, et al. 2015 ^74^ | 128 x 2-slice | 80/100kV | 370mA | Iopamidol (370mgI/ml) | 145+-35ml | 10 min | - |
| Ko, et al. 2007 ^76^ | 16-slice | 120kV | 310mA | Iopromide (370mgI/ml) | 100ml | 7 min | ± 20 mSv |
| Palmisano, et al. 2020 ^67^ | 128 x 2-slice | 80 kV (BMI<30) / 100 kV (BMI ≥ 30) | - | Iopromide (370mgI/ml) | 97-140ml | 10 min | - |
